# MicroRNA‐153‐3p increases autophagy in sevoflurane‐preconditioned mice to protect against ischaemic/reperfusion injury after knee arthroplasty

**DOI:** 10.1111/jcmm.15188

**Published:** 2020-04-02

**Authors:** Shuang Qiu, Benjuan Liu, Yanshuai Mo, Xueqin Wang, Lina Zhong, Xiao Han, Fuli Mi

**Affiliations:** ^1^ Department of Anesthesiology Linyi People's Hospital Linyi China

**Keywords:** autophagy, B‐cell lymphoma‐2, Beclin1, ischaemic/reperfusion injury, microRNA‐153‐3p, sevoflurane, total knee arthroplasty

## Abstract

The use of tourniquet during total knee arthroplasty (TKA) can result in ischaemia/reperfusion injury (IRI). Of interest, microRNAs (miRs) are reported to be involved in various kinds of IRI due to their ability in modulating autophagy. Therefore, the study aimed to investigate the effect of miR‐153‐3p on autophagy in IRI in vitro and in vivo under sevoflurane preconditioning. In the in vitro model, chondrocytes from naive mice were treated with 0% FBS alone or in combination with sevoflurane. Additionally, in vivo assays were conducted in mouse models with tourniquet‐induced IRI after TKA under or without sevoflurane preconditioning. The pathological observation in vivo validated that sevoflurane preconditioning protected the knee joint against IRI. Moreover, miR‐153‐3p expression was diminished in chondrocytes of the in vitro model and in cartilage tissue of the in vivo model, but its expression was appreciably up‐regulated in the presence of sevoflurane preconditioning. Mechanistic study showed that miR‐153‐3p disrupted the interaction between Bcl‐2 and Beclin1 by targeting Bcl‐2, thereby facilitating autophagy in chondrocytes under sevoflurane preconditioning. Furthermore, the experiments in human chondrocytes also verified the protective effects of miR‐153‐3p against IRI were realized through inhibiting Bcl‐2. Collectively, miR‐153‐3p overexpression blocks the interaction between Bcl‐2 and Beclin1 *via* down‐regulation of Bcl‐2 to promote autophagy of chondrocytes, thus protecting knee joint against IRI after TKA under sevoflurane preconditioning.

## 1 INTRODUCTION

The use of a tourniquet is employed in total knee arthroplasty (TKA) procedures in order to prevent blood loss.^1^ Furthermore, in the event a tourniquet is inflated, ischaemia will be induced to an extremity, the release of which results in subsequent ischaemia/reperfusion injury (IRI) to localized skeletal muscle, to systemic circulation and in some instances to vital distant organs such as the heart, kidneys, lungs and brain.^2^ IRI of limbs and distal organs induced by tourniquet has been reported by various studies following TKA.^3^ Similarly, accumulating studies continue to highlight IRI as an issue requiring further investigations in knee arthroplasty.^4^, ^5^, ^6^, ^7^ A previous study concluded that myocardial IRI could be repressed by means of sevoflurane‐increased autophagy.^8^ Sevoflurane has also been reported to attenuate IRI in certain organs including the heart, lung and liver in mice administered with sevoflurane, illustrated by a superior recovery following IRI.^9^, ^10^, ^11^, ^12^, ^13^ Additionally, a recent study demonstrated that the use of hyperbaric oxygen preconditioning confers neuro‐protective effects against IRI in neurocyte a process of which involves the induction of autophagy, highlighting the possible link between autophagy and protection against IRI following TKA.^14^

The ability of microRNAs (miRs) to regulate gene expression at a post‐transcriptional level has been shown to influence ischaemic preconditioning, ischaemic post‐conditioning and IRI.^15^ Additionally, miR‐153‐3p has been implicated in acute graft‐versus‐host disease.^16^ Furthermore, studies have indicated that B‐cell lymphoma‐2 gene (Bcl‐2) is directly targeted by miR‐153‐3p.^17^ As a suppressor of apoptosis, Bcl‐2 has been widely documented to promote cell survival and inhibits cell death.^18^ Furthermore, it has been observed that in human SGC‐7901 cells, Beclin1‐dependent autophagy is stimulated by small interfering RNA‐mediated Bcl‐2 silencing.^19^ Existing literature has revealed that the disrupted interaction between Beclin1 and Bcl‐2 arises following activation of AMPK which enhances autophagy and decreases cardiomyocyte apoptosis, which has been shown to be beneficial for the treatment of diabetic cardiomyopathy.^20^ Similarly, another study explored the contribution of the interaction between Beclin1 and Bcl‐2 induced by mammalian Ste20‐like kinase 1 (Mst1) and the suppression of autophagy.^21^ Notably, Peng et al provided evidence indicating that myocardial tissue is protected against IRI following ischaemic preconditioning (IPC) which blocks the interaction of Beclin1 and Bcl‐2 to increase autophagy.^22^ Based on the aforementioned studies, we hypothesized that miR‐153‐3p promotes the protection and alleviates the effects of IRI by mediating autophagy through the interaction between Beclin1 and Bcl‐2. Hence, the study was designed in order to investigate the effect of miR‐153‐3p on IRI following TKA as well as the underlying mechanism involved with Beclin1, Bcl‐2 and autophagy.

## 2 MATERIALS AND METHODS

### 2.1 Ethics statements

This study was performed with the approval of the Animal Committee of Linyi People's Hospital. Extensive efforts were made to minimize the suffering of the animals during our study.

### 2.2 Animals

A total of 66 male BL6/C57 mice (aged 7 weeks; weighing 21‐25 g) at specific pathogen‐free (SPF) grade were purchased from Shanghai SLAC Laboratory Animal Co., Ltd. Among the mice, 50 mice were subjected to TKA experiments, while the remaining mice were used to isolate primary chondrocytes.

### 2.3 Isolation and identification of primary chondrocytes

The mice were killed, after which their bilateral knee cartilages were obtained under aseptic conditions. The cartilages were washed with phosphate‐buffered saline (PBS) with the soft tissue as well as the bone tissues around the articular cartilages subsequently removed. The cartilages were treated with 2.5 g/L trypsin (Sigma‐Aldrich Chemical Company) for 15 minutes, with the excess tissue around the joint discarded. The cartilages were washed with PBS, cut into small blocks and detached with 0.1% type ΙΙ collagenase (Sigma‐Aldrich Chemical Company) for 4 hours. Next, the cell suspension was filtered using a 150‐mesh filter and centrifuged at 1000 rpm/min for 5 minutes. The supernatant was discarded, after which the cell precipitate was resuspended with Dulbecco's minimal essential medium/Ham's F12 (DMEM/F12, Thermo Fisher Scientific Inc) supplemented with 10% foetal bovine serum (FBS), 100 U/mL penicillin and 100 U/mL streptomycin. The cells were cultured in flask at 37°C with 5% CO_2_.

Collagen ΙΙ expression was measured by means of reverse transcription‐quantitative polymerase chain reaction (RT‐qPCR) on the 7th day of culture. The matrix secreted by chondrocytes was measured by Alcian blue staining in order to determine the cartilage characteristics. The primary chondrocytes were isolated and washed twice with PBS. Afterwards, the chondrocytes were fixed with 4% paraformaldehyde (Servicebio) for 20 minutes and stained with 1% Alcian blue (Servicebio) for 15 minutes. The chondrocytes were then washed with distilled water and observed under a microscope.

### 2.4 RT‐qPCR

The total RNA was extracted from cartilage tissues and primary chondrocytes using a Trizol RNA kit. The concentration of the RNA was subsequently determined using a NanoDrop 2000 spectrophotometer (Thermo Fisher Scientific Inc). The RNA was then reversely transcribed into cDNA using the ReverTra Ace^®^ qPCR RT Master Mix with gDNA Remover. The primers of miR‐153‐3p, collagen ΙΙ, Bcl‐2 and Beclin1 were designed and synthesized by Shanghai Sangon Biotechnology Co. Ltd., (Table 1), with glyceraldehyde‐3‐phosphate dehydrogenase (GAPDH) and U6 employed as the internal references. Fluorescence quantitative PCR was performed using the Bio‐Rad CFX96 Touch™ system based on the provided instructions of the 2× RealStar Green Mixture Kit (GenStar). The 2^−ΔΔCt^ method was applied to calculate the relative expression of the target genes. The experiment was repeated 3 times.

**TABLE 1:** Primer sequences for RT‐qPCR

Genes	Primer sequences
*Collagen ΙΙ‐F*	5′‐CAAGGAGAAGCCGGACA‐3′
*Collagen ΙΙ‐R*	5′‐AGCAGCTCCAGGGAATC‐3′
*miR‐153‐3p‐F*	5′‐ACACTCCAGCTGGGTTGCATAGTCACAAA‐3′
*miR‐153‐3p‐R*	5′‐CAGTGCGTGTCGTGGAGT‐3′
*Bcl‐2‐F*	5′‐AGGATGAAGTGCTCAGGTGC‐3′
*Bcl‐2‐R*	5′‐AGGATGAAGTGCTCAGGTGC‐3′
*Beclin1‐F*	5′‐GCTCCCGAGGTGAAGAGCAT‐3′
*Beclin1‐R*	5′‐GCCTGGGCTGTGGTAAGTAA‐3′
*U6‐F*	5′‐TCGCACAGACTTGTGGGAGAA‐3′
*U6‐R*	5′‐CGCACATTAAGCCTCTATAGTTACTAGG‐3′
*GAPDH‐F*	5′‐AGCTTGTCATCAACGGGAAG‐3′
*GAPDH‐R*	5′‐TTTGATGTTAGTGGGGTCTCG‐3′

Abbreviations: Bcl‐2, B‐cell lymphoma‐2; F, forward; GAPDH, glyceraldehyde‐3‐phosphate dehydrogenase; miR‐153‐3p, microRNA‐153‐3p; R, reverse; RT‐qPCR, reverse transcription‐quantitative polymerase chain reaction.

### 2.5 Animal treatment

Fifty mice were randomly selected to establish the IRI mouse model. After the mice had been killed, the bilateral femurs of mice were dissected, with 1 mm knee joint cartilage cut using a blade along the vertical direction of the long axis of the blade. The lateral section diameter of the femur was measured, after which a needle with a diameter of 0.2 mm was selected as a simple prosthesis.

The mice were anesthetized by intraperitoneal injection with 1% pentobarbital sodium (0.2 mL for each mice), fixed on an operating table and sterilized with conventional iodine and alcohol. The skin, subcutaneous tissue and joint capsule were cut in the middle of the knee to expose the knee joint. A longitudinal incision was then made to the joint capsule from the medial patella, with the right knee joint exposed by dislocating the patellar dislocation via knee flexion. An incision was made to the anterior cruciate ligament as well as the medial and lateral meniscus for tibial subluxation purposes. Next, osteotomy of tibia and femur was performed, after which bleeding was stopped using a tourniquet, with the pulp reamed accordingly. The model was then evaluated, with a simple prosthesis inserted into the joint after the prosthesis had been sterilized and disinfected under high‐temperature conditions. The deep fascia and skin were sutured in a layer by layer manner, after which the tourniquet was gradually deflated. After the wound had been covered with alcohol gauze, 200 U of gentamicin was injected into the muscle of the mice for three days (twice per day) after operation. Prior to tourniquet deflation, the modelled mice were permitted to inhale 2.4% sevoflurane (0426, Fuso Pharmaceutical Industries Ltd.) in a continuous manner until 30 minutes after suture.^23^, ^24^, ^25^ Among the sham‐operated mice, the right knee joint capsule was opened and closed under identical conditions. A total of 41 mice were successfully modelled, with a model success rate of 82%.

Six sham‐operated C57/BL6 mice were randomly selected and six IRI‐modelled mice that did not inhale sevoflurane. Besides, 24 IRI‐modelled mice inhaled sevoflurane and either remained untreated or were injected with inhibitor negative control (NC) + overexpression (oe)‐NC lentivirus, miR‐153‐3p inhibitor + oe‐NC lentivirus or miR‐153‐3p inhibitor + oe‐Bcl‐2 lentivirus or not (n = 6). On the third day post‐operation, the mice were punctured inward and backward in close proximity to the inferior pole of the patella via the lateral margin of the ligamentum patellae. After touching the prosthesis, lentiviruses were injected into the articular cavity with a dose of 1 × 10^6^ (50 μL). After 14 d of routine feeding, the tissues were collected for further examination.

### 2.6 Starvation and sevoflurane exposure of mouse primary chondrocytes

The primary chondrocytes were isolated from mice that had not been subjected to any operative procedures and cultured in a medium containing 10% FBS (control group). When cell confluence reached approximately 90%, the chondrocytes were passaged at a density of 2 × 10^5^ cells/dishes. The medium was replaced by a FBS free medium. A portion of the cells were then incubated at 37°C with 5% CO_2_ (0% FBS group), while the remaining portion of the cells were incubated at 37°C in a 5% CO_2_ incubator with sevoflurane gas introduced using an anaesthetic vaporizer (Drager Medical AG) at a rate of 2 L/min until the concentration of sevoflurane eventually reached 1%‐6% (0% FBS + sevoflurane group). The concentration of sevoflurane was monitored every minute using an anaesthesia analyser (Drager Medical AG) to ensure that the concentration was within the normal range. The chondrocytes were treated for 24 hours and used for subsequent experimentation.^26^

### 2.7 Human chondrocytes isolation and culture

Cartilage specimens collected from patients that had undergone operative procedures at our hospital were promptly washed with normal saline in order to remove any remaining blood and tissue fluids and stored at low‐temperature sterile conditions. After 3 rinses with PBS containing 1% penicillin‐streptomycin (100 U/mL penicillin and 100 μg/mL streptomycin), the degenerated cartilage tissue samples were minced into pieces of less than 1 mm^3^, followed by sequential detachment by 0.25% trypsin at 37°C for 30 minutes and by 0.2% collagenase II (prepared by DMEM containing 10% FBS and 1% penicillin‐streptomycin) under shaking at 37°C for 16 hours. Subsequently, chondrocytes were subjected to centrifugation, cultured in 5 mL DMEM culture solution with 20% PBS and constructed into cell suspension. The cells were then seeded into a culture flask at a density of 2.5 × 10^5^ cells/mL and cultured at 37°C in 5% CO_2_. After 48 hours, the culture medium was changed based on cell adherence. The growth rate and morphology of the cells were observed, recorded and photographed using an inverted microscope on a daily basis. The culture medium was changed regularly every 3 days.

### 2.8 Chondrocyte treatment

The primary chondrocytes from naive samples were seeded into a 6‐well plate. Confluent chondrocytes treated with 0% FBS+ sevoflurane, delivered with inhibitor NC, miR‐153‐3p inhibitor, oe‐NC plasmid or oe‐Bcl‐2 plasmid based on the instructions of the lipofectamine 2000 kit (Invitrogen). The plasmids, mimic and inhibitor as well as the reporting monomeric red fluorescent protein (mRFP)‐green fluorescent protein (GFP)‐light chain 3 (LC3) were all purchased from Sino Biological Inc. After transfection, the medium was renewed. After 48 hours, the chondrocytes were harvested for the following experiments. Each cellular experiment was confirmed by three independent biological replicates.

### 2.9 Haematoxylin‐eosin staining

On the 14th day post‐operation, the mice were killed, with the inferior 1/3 of the right femur as well as the superior 1/3 of the right tibia obtained, including the dense bone, bone marrow and cartilage tissues. The samples were embedded with paraffin and cut into sections for haematoxylin‐eosin (HE) staining purposes. The tissues were then washed 3 times with PBS and fixed with 4% paraformaldehyde overnight. Next, the tissues were washed 3 more times with PBS. The tissues were treated with the ethylenediaminetetraacetic acid (EDTA) decalcifying agent at 25°C and washed 3 times with PBS. The tissues were immersed in 70% alcohol overnight, in 80% and 90% alcohol for 1 hour, respectively, and 3 times in 100% alcohol (1 hour each time). Afterwards, the tissues were cleared twice with xylene (8 minutes each time), immersed in wax at 65°C (pre‐melting) for 3 hours and embedded in paraffin. A Leica LM2300 Paraffin Slicer was used to cut tissues into 5‐μm sections. HE staining methods and microscopic examination were performed in accordance with a previously reported method in literature.^27^

### 2.10 Flow cytometry

The chondrocytes were washed with pre‐cooled PBS and detached with trypsin without EDTA. The cells were then collected and subsequently washed twice with PBS. As per the instructions of the Annexin V‐FITC Apoptosis Detection Kit Ι (BD Pharmingen), the chondrocytes were resuspended in 500 μL of binding buffer and added with 5 μL fluorescein isothiocyanate (FITC) and 5 μL propidium iodide (PI) for incubation purposes for 15 minutes under conditions void of light. Apoptosis was measured using a flow cytometer (BD Pharmingen).

### 2.11 Co‐localization of Bcl‐2 and Beclin1

The chondrocytes were washed 3 times with PBS and fixed with 4% phosphonoformate (PFA) at room temperature for 30 minutes. The chondrocytes were washed 3 times with PBS, sealed with 1% bovine serum albumin (BSA) and incubated with primary antibodies (Abcam Inc) to anti‐rabbit Bcl‐2 and anti‐rabbit Beclin1 at 4°C overnight. The chondrocytes were then subjected to additional PBS washing, incubated with FITC‐labelled mouse anti‐rabbit immunoglobulin G (IgG) and CY3‐conjugated goat antimouse IgG antibodies (Santa Cruz Biotechnology Inc) at room temperature for 1 hour, followed by staining with 4',6‐diamidino‐2‐phenylindole (DAPI, Sigma‐Aldrich Chemical Company). The chondrocytes were mounted and observed under a fluorescence microscope.

### 2.12 Co‐immunoprecipitation (Co‐IP)

The chondrocytes were lysed with the cell lysis buffer containing protease inhibitor on ice for 30 minutes. The lysate was collected by centrifugation at 4°C for 30 minutes at 12 000 rpm. Part of the cell lysate was employed as the input. Another part of the cell lysate was incubated with 1 μg of anti‐Bcl‐2 antibody (Abcam Inc) or IgG (Abcam Inc) at 4℃ overnight. Afterwards, 10 μL of protein A agarose beads was washed 3 times with the cell lysis buffer. The pre‐treated protein A agarose beads were mixed with the antibody‐lysate mixture and incubated for a period of 2‐4 hours under 4°C conditions. The agarose beads were collected by means of centrifugation at 2664 *g* at 4°C for 3 minutes. The beads were then washed 3 times with 1 mL of cell lysis buffer. The concentration of the protein binding in the beads was determined and adjusted to an equal amount. The protein was mixed with an equal volume of 2× sodium dodecyl sulphate (SDS), and boiled for 5 minutes. The expression of Beclin1 was measured by Western blot analysis methods. The expression of Bcl‐2 binding to Beclin1 was investigated based on the aforementioned method, where the anti‐Beclin1 antibody (Abcam Inc) was utilized.

### 2.13 Western blot analysis

The chondrocytes were incubated with the RIPA lysis buffer (Beyotime Biotechnology Co., Ltd.) on ice for 5 minutes. The supernatant was collected with the protein concentration measured using a bicinchoninic acid (BCA) kit (Thermo Fisher Pierce). The proteins were then separated via sodium dodecyl sulphate‐polyacrylamide gel electrophoresis (SDS‐PAGE) with 4% concentrated gel and 10% concentrated gel and subsequently transferred onto a polyvinylidene fluoride (PVDF) membrane. The membrane was blocked with 5% skimmed milk powder for 1 hour and then incubated at 4°C overnight with the primary antibodies to P62 (1:100‐1:1000), Bcl‐2 (1:100‐1:1000), Beclin1 (1:1000), LC3 (1:3000) and GAPDH (1:5000). The membrane was washed with Tris‐buffered saline with Tween 20 (TBST) at room temperature and incubated with the horseradish peroxidase (HRP)‐labelled IgG antibody (Santa Cruz Biotechnology, Inc) at room temperature for 1 hour. The membrane was then washed 3 times with TBST and subsequently developed through the use of electrochemiluminescence (ECL) luminescent solution and photographed using the Bio‐Rad ChemiDoc™ imaging system. GAPDH was regarded as the internal reference with the relative expression of each protein calculated based on the ratio of the grey value of each protein to the grey value of GAPDH.

### 2.14 Dual‐luciferase reporter gene assay

The fragment containing the predicted binding site between miR‐153‐3p and 3’ untranslated region (3’UTR) of Bcl‐2 and the corresponding mutant fragment was inserted into the luciferase reporter vector (Beijing Huayueyang Biotechnology Co., Ltd.) in order to obtain the Bcl‐2‐wild‐type (WT) and Bcl‐2‐mutant‐type (MUT) reporter plasmids, respectively. The reporter plasmids were cotransfected with miR‐153‐3p mimic or the control plasmid into cells. Ranilla luciferase was employed as the internal reference. After 48 hours, the cells were collected and lysed. Luciferase reporter gene assay was performed on the dual‐luciferase reporter gene analysis system (Promega) based on the instructions of the luciferase assay kit (K801‐200, BioVision Research Products). The relative luciferase activity of the target reporter gene was calculated based on the ratio of the relative light units (RLU) of firefly luciferase to the RLU of the renilla luciferase.

### 2.15 Statistical analysis

Statistical analyses were conducted using SPSS 21.0 (IBM). Measurement data were expressed as mean ± standard deviation. If the data conformed to normal distribution and homogeneity of variances, the comparison between two groups in non‐paired design was analysed via non‐paired *t* test, and comparisons among multiple groups were performed by one‐way analysis of variance (ANOVA), followed by Turkey's post hoc test. *P* < .05 was considered to be indicative of statistical significance.

## 3 RESULTS

### 3.1 miR‐153‐3p is up‐regulated in IRI chondrocytes and mice pre‐treated with sevoflurane

**FIGURE 1:**
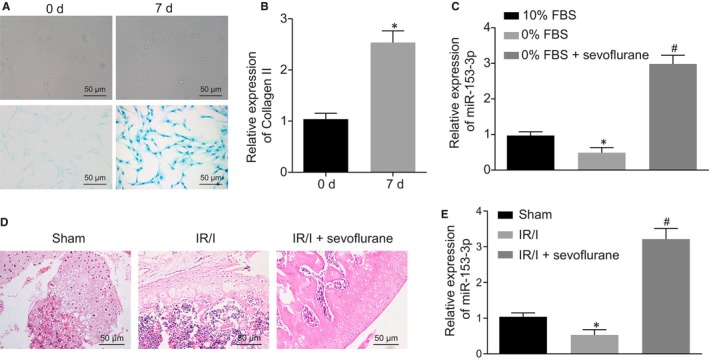
Sevoflurane preconditioning increases miR‐153‐3p expression in IRI chondrocytes and mice. A, Identification of isolated mouse primary chondrocytes by Alcian blue staining (200 ×). B, Expression of collagen ΙΙ in chondrocytes incubated at day 0 and day 7 determined by RT‐qPCR. C, Expression of miR‐153‐3p in chondrocytes treated with 10% FBS, 0% FBS and 0% FBS+ sevoflurane measured by RT‐qPCR. D, Pathological damage degree of sham‐operated mice, IRI mice and IRI mice treated with sevoflurane detected by HE staining (200 ×). E, Expression of miR‐153‐3p in sham‐operated mice, IRI mice and IRI mice treated with sevoflurane measured by RT‐qPCR. N = 6. *P < .05 vs chondrocytes incubated for 0 d, chondrocytes treated with 0% FBS, or IRI mice. ^#^ *P < *.05 vs IRI chondrocytes. Measurement data were expressed as mean ± standard deviation. The comparison between two groups was analysed by the non‐paired *t* test, and comparisons among multiple groups were performed by one‐way ANOVA, followed by Turkey's post hoc test. The cell experiment was repeated 3 times

The mouse primary chondrocytes were initially isolated and cultured, after which the matrix secreted by the chondrocyte was stained with Alcian blue in order to confirm the chondrocyte characteristics. RT‐qPCR was conducted to determine collagen ΙΙ expression in the chondrocytes. As depicted in Figure 1A‐B, after 7 days of incubation, the secretion of extracellular matrix as well as the expression of collagen ΙΙ was increased (*P* < .05), which was indicative of successful isolation of the chondrocytes. The primary chondrocytes from naive mice were cultured with either a medium containing 10% FBS, 0% FBS or sevoflurane. Diminished expression of miR‐153‐3p was detected in 0% FBS‐treated chondrocytes when compared to 10% FBS‐treated chondrocytes (*P* < .05). Sevoflurane treatment led to an elevated miR‐153‐3p expression in the presence of 0% FBS (Figure 1C).

After the IRI mouse model post‐TKA had been established, the IRI mice were either treated or not treated with sevoflurane through inhalation during modelling. Following completion of the operative procedures, swelling of the entire knee joint as well as a high skin temperature was identified in the IRI mice, with a significant degree of white gelatinous fluid detected in the articular cavity and around the prosthesis as well as the swollen synovium. HE staining revealed a large number of inflammatory cell infiltration around the prosthesis and surrounding tissues, with marked fibrous tissue proliferation, small vessel dilatation and congestion. Among the sevoflurane‐treated IRI mice, distinct signs of joint hyperosteogeny were detected, with no signs of high skin temperature detected, and a small amount of transparent fluid identified in the articular cavity and around the prosthesis. Additionally, a less significant degree of inflammatory cell infiltration, fibrous tissue proliferation, and haemorrhage around the prosthesis were detected (Figure 1D). The expression of miR‐153‐3p was decreased in cartilage tissues of IRI mice when compared to the sham‐operated mice (*P* > .05), while higher levels were identified in the IRI mice treated with sevoflurane (*P* < .05; Figure 1E). Taken together, the use of tourniquet led to IRI during TKA, which could be alleviated by sevoflurane preconditioning. Furthermore, the expression of miR‐153‐3p was elevated following sevoflurane preconditioning during TKA, highlighting the potential crucial role of miR‐153‐3p in protecting against IRI.

### 3.2 Bcl‐2 is a target gene of miR‐153‐3p and down‐regulated in IRI chondrocytes and mice pre‐treated with sevoflurane

**FIGURE 2:**
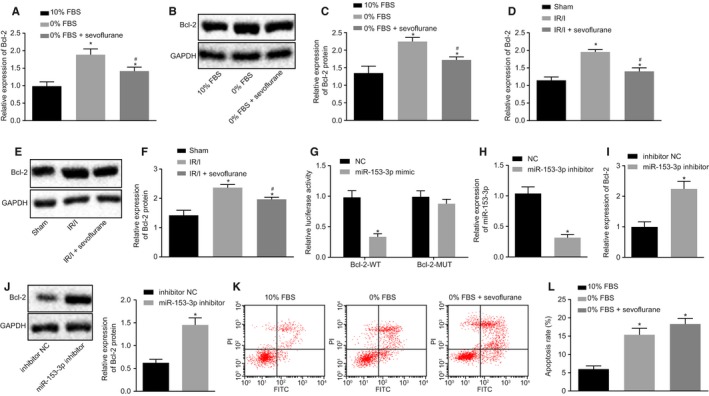
miR‐153‐3p is involved in sevoflurane preconditioning‐induced protection against IRI by down‐regulating Bcl‐2. A, Bcl‐2 mRNA expression in chondrocytes treated with 10% FBS, 0% FBS or 0% FBS+ sevoflurane determined by RT‐qPCR. B, C, Bcl‐2 protein expression in chondrocytes treated with 10% FBS, 0% FBS and 0% FBS+ sevoflurane determined by Western blot analysis. D, Bcl‐2 mRNA expression in sham‐operated mice, IRI mice and IRI mice treated with sevoflurane measured by RT‐qPCR. E, F, Bcl‐2 protein expression in sham‐operated mice, IRI mice and IRI mice treated with sevoflurane determined by Western blot analysis. N = 6. G, The targeting relationship Bcl‐2 and miR‐153‐3p assessed by the dual‐luciferase reporter gene assay. H, Transfection efficacy of miR‐153‐3p inhibitor in IRI mice treated with sevoflurane determined by RT‐qPCR. I, Bcl‐2 mRNA expression in IRI mice treated with sevoflurane and miR‐153‐3p inhibitor measured by RT‐qPCR. J, Bcl‐2 protein expression in IRI mice treated with sevoflurane and miR‐153‐3p inhibitor determined by Western blot analysis. N = 6. K, L, cell apoptosis in chondrocytes treated with 10% FBS, 0% FBS or 0% FBS+ sevoflurane evaluated by flow cytometry. *P < .05 vs chondrocytes treated with 10% FBS, sham‐operated mice, NC treatment or IRI mice treated with sevoflurane and inhibitor NC; ^#^ *P* < .05 vs chondrocytes treated with 0% FBS or IRI mice. Measurement data were expressed as mean ± standard deviation. Comparison between two groups was analysed by non‐paired *t* test, and comparisons among multiple groups were performed by one‐way ANOVA, followed by Turkey's post hoc test. The cell experiment was repeated 3 times

Higher expression levels of Bcl‐2 were identified both in vivo (in the primary chondrocytes of IRI mice with different treatment) and in vitro (in the primary chondrocytes of naive mice). The expression of Bcl‐2 was increased in the chondrocytes treated with 0% FBS and sevoflurane when compared with the chondrocytes treated with 10% FBS. Meanwhile, the expression of Bcl‐2 in chondrocytes treated with 0% FBS was higher than that in chondrocytes treated with 0% FBS and sevoflurane (*P* < .05; Figure 2A‐C). Moreover, the expression of Bcl‐2 was lower in both the IRI mice that had been treated with or without sevoflurane when compared to the sham‐operated mice (*P* < .05). Compared with IRI mice, IRI mice treated with sevoflurane had decreased Bcl‐2 expression (*P* < .05; Figure 2D‐F). It was presumed that there was the binding site between Bcl‐2 and miR‐153‐3p in the RNA22 database. Dual‐luciferase reporter gene assay was subsequently performed in order to determine whether Bcl‐2 was indeed a target gene of miR‐153‐3p. The results obtained revealed that luciferase activity of Bcl‐2‐WT was reduced by miR‐153‐3p mimic when compared with NC treatment (*P* < .05), while no significant difference was detected regarding the luciferase activity of Bcl‐2‐MUT by miR‐153‐3p mimic (*P* > .05; Figure 2G). Our results provided evidence verifying that miR‐153‐3p could bind to Bcl‐2. Meanwhile, the treatment of miR‐153‐3p inhibitor diminished the expression of miR‐153‐3p (Figure 2H) while acting to increase the expression of Bcl‐2 (Figure 2I‐J) in the IRI mice in the presence of sevoflurane. Besides, compared to 10% FBS treatment, the treatment of 0% FBS or sevoflurane elevated the apoptosis of chondrocytes (*P* < .05). However, there was no notable difference detected in relation to the rate of apoptosis between the chondrocytes treated with 0% FBS‐treated and chondrocytes treated with 0% FBS and sevoflurane (*P* > .05; Figure 2K‐L). The aforementioned results highlighted that miR‐153‐3p was a key factor in the protection mechanism of sevoflurane preconditioning. The overexpression of miR‐153‐3p triggered a decline in the expression of Bcl‐2, while down‐regulation of Bcl‐2 failed to influence the apoptosis of chondrocytes. Based on our results, we concluded that miR‐153‐3p might confer protection against IRI via a different mechanism.

### 3.3 miR‐153‐3p disrupts the interaction between Bcl‐2 and Beclin1 by targeting Bcl‐2 to promote chondrocyte autophagy in vitro

**FIGURE 3:**
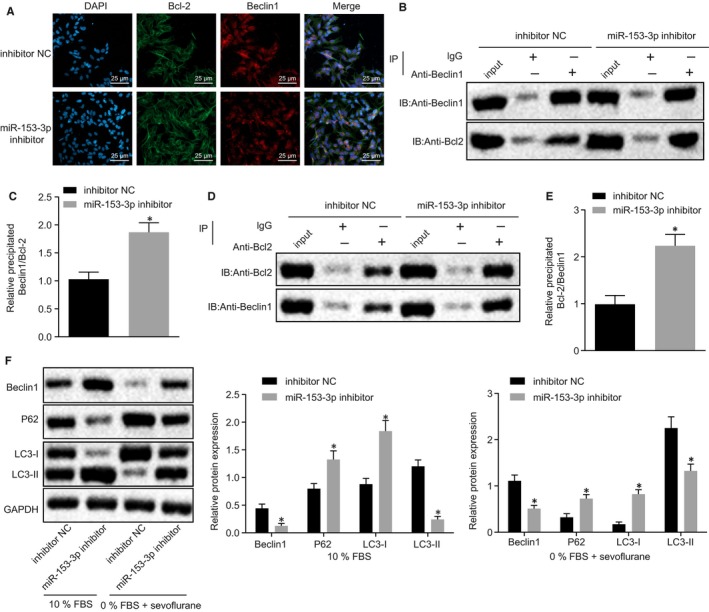
miR‐153‐3p induces mouse chondrocyte autophagy by blocking the interaction between Bcl‐2 and Beclin1 in vitro. A, The co‐localization of Bcl‐2 and Beclin1 after inhibition of miR‐153‐3p in chondrocytes treated with 0% FBS+ sevoflurane (400 ×). B, C, Bcl‐2 protein pulled down by Beclin1 after inhibition of miR‐153‐3p in chondrocytes treated with 0% FBS+ sevoflurane measured by Co‐IP. Anti‐Beclin1 was the pull‐down antibody, and input was control. D, E, Beclin1 protein pulled down by Bcl‐2 after inhibition of miR‐153‐3p in chondrocytes treated with 0% FBS+ sevoflurane measured by Co‐IP. Anti‐Bcl‐2 was the pull‐down antibody, and input was control. F, Expression of Beclin1 and autophagy‐related factors (P62, LC3‐I and LC3‐II) after inhibition of miR‐153‐3p in chondrocytes treated with 0% FBS+ sevoflurane measured by Western blot analysis. *P < .05 vs chondrocytes treated with 0% FBS+ sevoflurane +inhibitor NC. Measurement data were expressed as mean ± standard deviation. The comparison between two groups was analysed by non‐paired *t* test. The cell experiment was repeated 3 times

After the chondrocytes had been treated with miR‐153‐3p inhibitor in the presence of 0% FBS+ sevoflurane, the co‐localization of Bcl‐2 and Beclin1 was assessed accordingly. The results obtained indicated that following miR‐153‐3p inhibition, the co‐localization areas of Bcl‐2 and Beclin1 were both markedly increased (Figure 3A). Additionally, Co‐IP depicted that inhibition of miR‐153‐3p increased Bcl‐2‐bound Beclin1 protein (Figure 3B‐E). Western blot analysis was performed in order to determine the expression of Beclin1 and autophagy‐related factors (P62, LC3‐I and LC3‐II). As depicted in Figure 3F, when miR‐153‐3p was inhibited in chondrocytes treated with 0% FBS+ sevoflurane, P62 and LC3‐I expression was increased while the expression of LC3‐II was decreased, indicating the decrease of autophagy. In conclusion, in regard to the protection mechanism of sevoflurane preconditioning, miR‐153‐3p silencing promotes the interaction between Bcl‐2 and Beclin1 to inhibit chondrocyte autophagy.

### 3.4 miR‐153‐3p alleviates IRI by promoting chondrocyte autophagy in vivo

**FIGURE 4:**
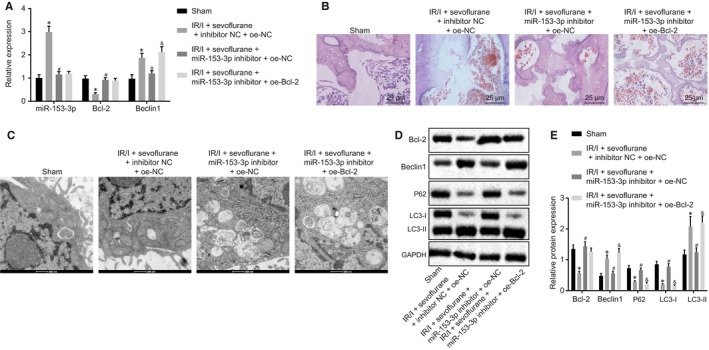
miR‐153‐3p attenuates IRI through the induction of autophagy of mouse chondrocytes in vivo. A, Expression levels of Bcl‐2, Beclin1, miR‐153‐3p in sham‐operated mice and IRI mice treated with sevoflurane + inhibitor NC + oe‐NC, sevoflurane + miR‐153‐3p inhibitor + oe‐NC and sevoflurane + miR‐153‐3p inhibitor + oe‐Bcl‐2 measured by RT‐qPCR. B, The morphology and pathological changes of cartilage tissues determined by HE staining (400 ×). C, The autophagosomes of cartilage tissues observed under the electron microscope (2000 ×). D‐E, Expression levels of Bcl‐2, Beclin1 and autophagy markers (P62, LC3‐I and LC3‐II) in cartilage tissues of sham‐operated mice and IRI mice treated with sevoflurane + inhibitor NC + oe‐NC, sevoflurane + miR‐153‐3p inhibitor + oe‐NC and sevoflurane + miR‐153‐3p inhibitor + oe‐Bcl‐2 measured by Western blot analysis. N = 6. *P < .05 vs sham‐operated mice; ^#^ *P* < .05 vs IRI mice treated with sevoflurane, inhibitor NC and oe‐NC; ^&^ *P* < .05 vs IRI mice treated with sevoflurane, miR‐153‐3p inhibitor and oe‐NC. Measurement data were expressed as mean ± standard deviation. The comparisons among multiple groups were performed by one‐way ANOVA, followed by Turkey's post hoc test

The IRI mice pre‐treated with sevoflurane were established, and the joint was injected with oe‐NC, inhibitor NC, miR‐153‐3p inhibitor or oe‐Bcl‐2. On the 14th day after operation, the morphological and pathological changes, number of autophagosomes, expression levels of Bcl‐2, Beclin1, miR‐153‐3p as well as the autophagy markers (P62, LC3‐I and LC3‐II) in the cartilage tissues of IRI mice were evaluated after different treatment. The results obtained exhibited that sevoflurane preconditioning increased expression of miR‐153‐3p (Figure 4A), Beclin1 and LC3‐II (Figure 4A,D) but decreased expression of Bcl‐2, P62 and LC3‐I (Figure 4A,D) in IRI mice, as well as increased autophagy in cartilage tissues (Figure 4C). Our results indicated that after sevoflurane preconditioning in IRI mice, miR‐153‐3p was up‐regulated which stimulated chondrocyte autophagy. Moreover, after miR‐153‐3p was inhibited, decreased expression of Beclin1 and LC3‐II and increased expression of Bcl‐2, P62 and LC3‐I (Figure 4A,D) were observed in IRI mice, with decreased autophagosomes in cartilage tissues (Figure 4C) and deteriorated cartilage damages (Figure 4B). These results revealed that after sevoflurane preconditioning in IRI mice, inhibiting miR‐153‐3p could reduce the autophagy of chondrocytes, a feature of which is not conducive to post‐injury repair. The up‐regulation of Bcl‐2 increased chondrocyte autophagy (Figure 4A,C,D) and promoted post‐injury repair (Figure 4B). Altogether, our results suggested that miR‐153‐3p promoted chondrocyte autophagy which alleviated IRI in vivo.

### 3.5 miR‐153‐3p promotes sevoflurane‐induced autophagy in human chondrocytes by mediating Bcl‐2

**FIGURE 5:**
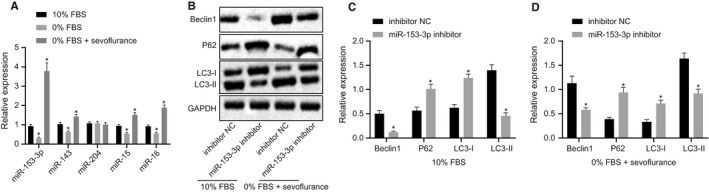
miR‐153‐3p promotes sevoflurane‐induced autophagy in human chondrocytes by mediating Bcl‐2. A, Expression of miR‐153‐3p, miR‐143, miR‐204, miR‐15 and miR‐16 in human chondrocytes detected by RT‐qPCR after treatment of 10% FBS, 0% FBS and sevoflurane. B, Expression of Beclin1 and autophagy‐related proteins P62, LC3‐1 and LC3‐II in human chondrocytes treated by 10% FBS, 0% FBS and sevoflurane were determined by Western blot analysis after depletion of miR‐153‐3p. C, Protein bands of Beclin1 and autophagy‐related proteins P62, LC3‐1 and LC3‐II in human chondrocytes treated by 10% FBS after depletion of miR‐153‐3p. D, Protein bands of Beclin1 and autophagy‐related proteins P62, LC3‐1 and LC3‐II in human chondrocytes treated by 0% FBS and sevoflurane after depletion of miR‐153‐3p. *P < .05 vs human chondrocytes treated with 10% FBS or inhibitor NC

After human chondrocyte starvation, the expression of miR‐153 was decreased, while sevoflurane preconditioning was found to elevate miR‐153 expression (Figure 5A). Previous research has indicated that miR‐143,^28^ miR‐204,^28^ miR‐15 and miR‐16^29^, ^30^ are all capable of regulating the expression of Bcl‐2. The Western blot analysis results revealed that miR‐153 could regulate autophagy in human chondrocytes and that Bcl‐2 expression was suppressed by miR‐153 (Figure 5B‐D). The aforementioned results suggested that miR‐153 could promote autophagy induced by sevoflurane in human chondrocytes by inhibiting the expression of Bcl‐2.

## 4 DISCUSSION

Ischaemic/reperfusion injury has been widely documented to not only inflict injury at the site of ischaemia, but can also lead to a cascade of damage to distal and non‐ischaemic organs.^31^ The use of tourniquet in TKA has been speculated to contribute to IRI in localized skeletal muscle, systemic circulation as well as vital distant organs.^2^ The emerging role of autophagy in various cardiovascular diseases, including that of heart failure, dilated cardiomyopathy and ischaemic heart disease has been discussed in many reports.^32^ Moreover, a previous study revealed the existence of a correlation between miRs with autophagy and upstream autophagy signalling pathways, including p53, AMPK and Bcl‐2.^33^ Herein, the current study was performed with the overall objective of elucidating the role of miR‐153‐3p in the protective mechanism associated with sevoflurane preconditioning in IRI, both in vivo and in vitro. Subsequently, our findings demonstrated that miR‐153‐3p promoted autophagy in IRI models in vivo and in vitro by means of blocking the interaction between Bcl‐2 and Beclin1 under sevoflurane preconditioning.

Sevoflurane preconditioning elevated autophagy in IRI mice, an observation of which was accompanied by up‐regulated LC3‐II and decreased P62 and LC3‐I, highlighting the link between autophagy promotion and the alleviation of IRI following TKA. A previous report suggested that P62, as an autophagy marker, is negatively correlated with autophagy.^34^ LC3‐II is an autophagosome marker in mammals and has been generally applied as an investigative tool for the role of autophagy in bacterial and viral infections, tumorigenesis as well as neurodegenerative and neuromuscular diseases.^35^ LC3‐I can be bind to phosphatidylethanolamine to form LC3‐II which is accumulated to autophagosomal membranes.^36^ A recent study demonstrated that IRI was suppressed by increased neurocyte autophagy induced by hyperbaric oxygen preconditioning, a finding of which was consistent with the results of the current study.^14^ Similarly, existing literature has demonstrated that remote limb ischaemic post‐conditioning confers cardio protection against myocardial IRI in normal mice by inducing autophagy.^37^

Furthermore, a key finding of the current study illustrated that miR‐153‐3p overexpression promotes autophagy by means of disrupting the interaction between Bcl‐2 and Beclin1 in IRI mice following TKA. It is well acknowledged that Beclin1 is involved at various stages of myocardial IRI.^38^ Tang et al concluded that HBV × protein increases autophagy by increasing Beclin1 expression.^39^ Moreover, a previous study revealed promoted autophagy in N2a with IRI after melatonin treatment, accompanied by an elevation in the expressions of Beclin1 and LC3‐II expression.^40^ Bcl‐2 has been proven to interact with Beclin1 and contribute to the suppression of Beclin1‐dependent autophagy.^41^ Du et al revealed that Bcl‐2 silencing promotes Beclin1‐dependent autophagy in gastric cancer cells.^19^ Disruption of the interaction between Bcl‐2 and Beclin1 has been implicated in maslinic acid‐promoted autophagy in rat pheochromocytoma cells.^42^ Another study indicated that autophagy is induced by blocking the interaction between Bcl‐2 and Beclin1, as a result of BH3 mimetic S1 promoting autophagy via blockade of Bcl‐2/Beclin1 interaction in human glioma cells.^43^ More importantly, a previous investigation demonstrated that Bcl‐2 was directly targeted and negatively regulated by miR‐153‐3p.^17^

**FIGURE 6:**
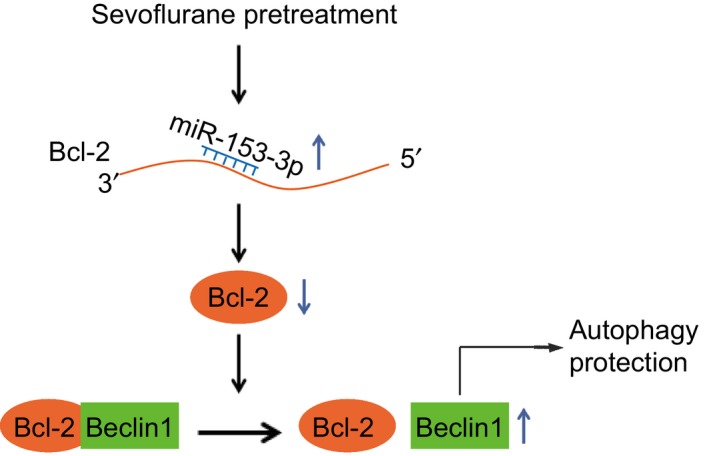
The mechanism of miR‐153‐3p on autophagy via Bcl‐2 and Beclin1 in IRI mice after TKA under sevoflurane preconditioning. Sevoflurane preconditioning increased miR‐153‐3p expression which targeted Bcl‐2 and down‐regulated Bcl‐2 to disrupt the interaction between Bcl‐2 and Beclin1, ultimately elevating autophagy and alleviating IRI following TKA

In conclusion, the findings of our study present in vivo and in vitro evidence supporting the notion that miR‐153‐3p alleviates IRI in sevoflurane pre‐treated chondrocytes and mice following TKA, via the induction of autophagy by blocking the interaction between Bcl‐2 and Beclin1 (Figure 6). Our findings highlight the potential of miR‐153‐3p up‐regulation as a potential clinically viable target capable of enhancing the protection of sevoflurane preconditioning against IRI following TKA. The potential role of sevoflurane in regulation of Beclin1 was not fully explored. Therefore, more detailed experiments are needed in the future to confirm the findings of our study.

## CONFLICT OF INTEREST

None.

## AUTHOR CONTRIBUTIONS

Shuang Qiu, Benjuan Liu and Xueqin Wang designed the study. Lina Zhong and Xiao Han collated the data, Fuli Mi and Yanshuai Mo contributed to drafting the manuscript. All authors have read and approved the final submitted manuscript.

## ETHICAL APPROVAL

This study was performed with the approval of the Animal Committee of Linyi People's Hospital. Extensive efforts were made to minimize the suffering of the animals during our study.

## Data Availability

The data used to support the findings of this study are available from the corresponding author upon request.
